# Emerging Lung Cancer Therapeutic Targets Based on the Pathogenesis of Bone Metastases

**DOI:** 10.1155/2014/236246

**Published:** 2014-08-14

**Authors:** Moses O. Oyewumi, Adnan Alazizi, Daniel Wehrung, Rami Manochakian, Fayez F. Safadi

**Affiliations:** ^1^Department of Pharmaceutical Sciences, College of Pharmacy, Northeast Ohio Medical University, Rootstown, OH 44272, USA; ^2^Division of Hematology/Oncology, Department of Medicine, Case Western Reserve University, Louis Stokes Veteran Affairs Medical Center, Cleveland, OH 44106, USA; ^3^Department of Anatomy and Neurobiology, College of Medicine, Northeast Ohio Medical University, Rootstown, OH 44272, USA

## Abstract

Lung cancer is the second most common cancer and the leading cause of cancer related mortality in both men and women. Each year, more people die of lung cancer than of colon, breast, and prostate cancers combined. It is widely accepted that tumor metastasis is a formidable barrier to effective treatment of lung cancer. The bone is one of the frequent metastatic sites for lung cancer occurring in a large number of patients. Bone metastases can cause a wide range of symptoms that could impair quality of life of lung cancer patients and shorten their survival. We strongly believe that molecular targets (tumor-related and bone microenvironment based) that have been implicated in lung cancer bone metastases hold great promise in lung cancer therapeutics. Thus, this paper discusses some of the emerging molecular targets that have provided insights into the cascade of metastases in lung cancer with the focus on bone invasion. It is anticipated that the information gathered might be useful in future efforts of optimizing lung cancer treatment strategies.

## 1. Introduction

Lung cancer is a significant public health burden in the USA. It is estimated that, in 2014, there will be approximately 224,000 newly diagnosed cases of lung cancer and 159,000 deaths from lung cancer [1]. There are two main subcategories of lung cancer: nonsmall cell lung cancer (NSCLC) and small cell lung cancer (SCLC). The majority of cases (85%) can be classified as NSCLC while about 15% of cases are of SCLC type. There are three main subtypes of NSCLC which are adenocarcinoma (~40% of cases), squamous cell carcinoma (~25–30% of cases), and large-cell carcinoma (~10–15% of cases).

It is generally believed that the high mortality rate of lung cancer cases may be a result of the aggressiveness and invasive and metastatic potential of the disease and the fact that it is not easily detectable until it reaches advanced stages [2]. In fact, over two-thirds of lung cancer patients have regional lymph-node involvement or distant metastases at the time of presentation [3]. The overall 5-year survival rate for NSCLC is still at 15%. The prognosis is dependent on the stage of the disease and extent of metastases. For instance, the five-year survival rate for patients with (stage IA) after surgical resection is close to 70% but the five-year survival rate for patients with stage IV (metastatic disease) is as low as 2% [4]. SCLC is characterized by many features that connote more aggressive nature than NSCLC with fast growth rates and early hematogenous spread with associated 5-year survival rate of 5–10% [5]. Overall, it is widely accepted that tumor metastasis is a major barrier to treatment of lung cancer [6]. In spite of the advances in treatment of primary malignancy, cases of relapse and metastatic spread have created major hurdles to reaching the desired treatment outcome [7].

The bone is one of the most common metastatic sites for lung cancer [8, 9]. Involvement of bone in lung cancer metastases is a major source of morbidity and mortality arising from skeletal related events (SREs) [10]. Common complications include intractable pain, bone destruction, hypercalcemia, nerve compression syndromes, and pathological fractures [10, 11]. Bone metastasis is a major determinant of treatment outcome, quality of life, and survival for lung cancer patients [12–14]. Certainly, the presence of bone metastasis usually leads to worse outcome and shorter median survival [11, 15, 16].

Although there are currently some available palliative treatment options for patients with bone metastases from any solid tumor origin such as radiation therapy and bone targeted/bone strengthening treatments (bisphosphonates and denosumab) that are used in clinical practice, none of them is specific for lung cancer metastases and they do not affect the poor survival outcome of this disease. This underscores the need to assess molecular targets that can be exploited in prevention or clinical management of lung cancer metastases to bone. We believe that these molecular targets could guide in determining timing of therapeutic interventions that offer the best opportunity to prevent and/or halt extent of bone metastases. Thus, in this report, we will assess the data on lung cancer bone metastases with the intention of evaluating key molecular targets that could be applied in optimizing lung cancer treatment strategies.

## 2. Pathogenesis of Lung Cancer Metastasis to Bone

Metastases of lung cancer cells to bone are achieved through a complex cascade of events which can be broadly depicted as follows [6, 17]: (a) tumor cell detachment from the primary site and invasion through the basement membrane and stroma, (b) intravasation into lymphatic system or blood vessels, (c) survival of tumor cells within the circulation and plantation at distant sites, (d) tumor cells extravasation into distant tissue microenvironment, (e) existence of tumor cells in the distant tumor stroma, and (f) proliferation to micrometastases and formation of tumor at bone metastatic sites.

It is widely accepted that each step involved in lung cancer metastases presents multiple opportunities to halt lung cancer cell progression from the primary site and/or hinder survival and expansion at the metastatic sites [6]. For instance, invasion into distant sites will require degradation of extracellular matrix components (collagen IV, laminin, and fibronectin) which is accomplished most likely by matrix metalloproteinase. Also, cancer cell adhesion has also been implicated in the metastatic process involving integrins as adhesion molecules that are involved in cell-matrix and cell-cell interactions. It was reported that tumor cells that express *α*_4_ *β*_1_ or *α*_2_ *β*_1_ integrins may preferentially adhere to bone [18].

**Figure 1:**
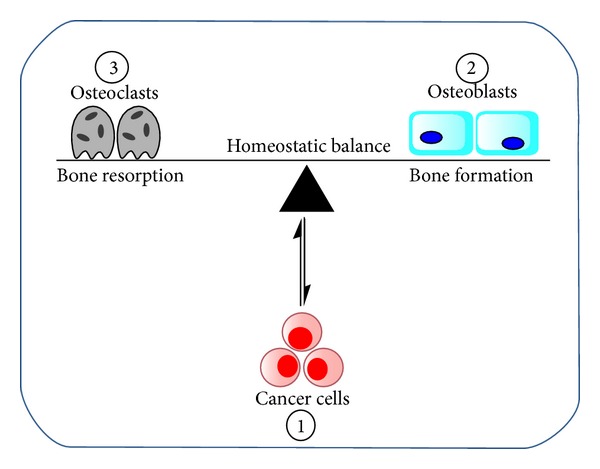
Schematic of lung cancer invasion of bone microenvironment resulting in disruption of normal bone homoeostasis. The invading tumor cells (1) primarily influence the functions of osteoclasts (2) and osteoblasts (3) that are involved in regulating bone modeling.

The ability of lung cancer cells to invade the bone has been broadly illustrated by the seed and soil theory in which case the bone provides a fertile environment (soil) for cancer cell (seed) to inhabit and grow [19]. It is clear from many other reports that the seed and soil theory is only a partial representation or an oversimplification of bone metastasis cascade [19]. The bone is a dynamic tissue that is continually undergoing remodeling throughout life via intricate functions of bone-resorbing osteoclasts and bone-forming osteoblasts [20]. The normal bone homeostasis is achieved by initiation of a normal bone remodeling cycle, which begins with the recruitment of osteoclast precursor cells. Subsequently, the osteoclast precursor cells differentiate into mature osteoclasts that later synthesize and release proteolytic enzymes that digest the collagen matrix [21]. The bone resorption as the first phase of the remodeling cycle is regulated by apoptosis of osteoclasts. During the second phase of the remodeling cycle, preosteoblasts are attracted to mesenchymal stem cells in the bone marrow. Bone formation is achieved by mature osteoblasts that synthesize the bone matrix and regulate the mineralization of the newly formed bone. Eventually, some of the mature osteoblasts may be trapped within mineralized bone and become osteocytes. Any interference with the normal bone homeostasis leading to a higher bone formation activity will result in a net increase in bone mass whereas a higher bone resorption activity will result in a net loss of bone mass (Figure 1). The mechanism for the development of lung cancer bone metastasis is not fully understood; but insights into how the bone can harbor tumor cells leading to distortion of the normal bone remodeling activities have been useful in identifying some intriguing therapeutic targets. It is generally believed that the bone is a favored metastatic site for many reasons, which include [11, 22–24] (i) high blood flow especially to the red marrow coupled with abundant sinusoids, (ii) sluggish blood flow in the metaphysis facilitating intimate interaction between endothelium and tumor cells, (iii) a large source of immobilized growth factors (such as transforming growth factor, insulin-like growth factors, fibroblast growth factors, platelet-derived growth factors, bone morphogenetic proteins, and calcium), and (iv) continuous and dynamic turnover of bone matrix that can unlock vast resources (cytokines and growth factors) that are needed for tumor survival. Many investigators have represented the pathogenesis of bone metastasis as a vicious cycle that is based on the crosstalk between tumor cells and bone microenvironment leading to disruption of normal bone homeostasis that eventually fuels tumor growth [23]. While this report focuses on metastasis of lung cancer to bone, there are major differences between metastatic bone cancers and cancers that originate in the bone (primary malignant bone cancers). The most prevalent primary malignant bone cancer is osteosarcoma which is often diagnosed in children and adolescents during growth spurts [25]. Other forms of primary bone cancers are chondrosarcoma and Ewing sarcoma. Primary bone cancers most often target the long bones and the joints in the arms and legs whereas metastatic cancer often spreads to bones near the middle of the body such as the spine, pelvis, upper leg bones, upper arm bones, and ribs [26].

## 3. Molecular Targets Implicated in Lung Cancer Bone Metastasis

Tumor metastases to bone can be classified as osteolytic (bone destruction), osteoblastic (abnormal bone formation), or mixed of osteolytic and osteoblastic [14]. In lung cancer, osteolytic metastases are the most common type [7, 10, 27]. The classification is an indication of the interactions between tumor cells and bone cellular elements (osteoclasts and osteoblasts). The osteoclasts (derivatives of the pluripotent hematopoietic precursors in the marrow) are the primary cells involved in tumor-mediated osteolysis. Osteoclast differentiation and maturation are the critical steps in lung cancer metastases to bones [17]. It has been reported that lung cancer cells are characterized by distinct cytokine profile and growth factors [16, 28]. These include parathyroid hormone-related peptide (PTHrP), IL-1, IL-7, receptor activator of nuclear factor-*κ*B ligand (RANKL), and tumor necrosis factor (TNF-*α*) which are involved in the stimulation of osteoclast differentiation and activation. The prognostic significance of bone markers in patients with lung metastasis to bone has been evaluated [15]. The investigators observed elevated levels of most bone formation markers (bone alkaline phosphatase, osteocalcin, and osteoprotegerin) and bone absorption markers (urinary calcium, osteopontin, and RANKL) [15]. Several reports have demonstrated the therapeutic implications of the receptor activator of nuclear factor- (NF-) *κ*B (RANK), its ligand RANKL, and the protein osteoprotegerin (OPG) [29]. RANKL is a membrane-bound protein expressed primarily on the surface of osteoblasts and bone marrow stroma cell [30, 31]. Upon binding to RANK on the surface of osteoclast precursors, RANKL will stimulate osteoclast differentiation and maturation. Another interesting protein in the OPG/RANK/RANKL axis is OPG, which is a decoy receptor of RANKL that is produced by osteoblast/stromal cells [12, 32, 33]. OPG can prevent bone destruction by blocking the binding of RANKL to RANK, thereby inhibiting osteoclast differentiation and activation [33]. Previous studies have shown that RANKL plays a critical role in osteolytic lesions in bone [34–36], which provides the basis for blocking RANKL-RANK interaction in order to halt osteolytic lesions. Other findings that substantiated the role of RANKL-RANK in lung cancer progression and bone metastasis include (i) the demonstration that RANKL was effective in triggering the migration in human cancer cells that express RANK [32] and (ii) RANK-Fc (a recombinant soluble protein consisting of extracellular domain of RANK coupled with the Fc domain of human IgG) was effective in inhibiting the RANK-RANKL interaction, thereby preventing osteoclastogenesis [37]. Dysregulation of RANKL/RANK/OPG system has been detected in several tumors including lung cancer, which has afforded an interesting target for therapeutic intervention [32, 38, 39]. In this regard, denosumab is the first RANKL inhibitor approved by Food and Drug Administration (FDA) for clinical management of cases of bone metastases. In metastatic cancers involving the bone, denosumab has been shown to suppress markers of bone resorption [40–42]. Although bone metastatic lesions from lung cancer invasion are mainly osteolytic [24, 43], cases of mixed lesions have been reported which underscores the need for therapeutic strategies that target both osteolytic and osteoblastic components of bone colonization. Many investigators have reported that blocking osteolytic activity is important even when treating osteoblastic lesions [14]. A major reason is that every primary or metastatic cancer in bone begins with osteolysis [11, 44], possibly to fuel the vicious cycle that supports tumor growth at the bone metastatic sites. The molecular basis of osteoblastic lesion that could occur in mixed lung cancer bone lesions has provided useful information. The primary bone cells in osteoblastic activity are osteoblasts which are involved in forming woven bone, common feature of osteoblastic metastases [12, 18, 45]. In osteoblastic bone metastases, patients suffer severe bone pain and poor quality of life with high predisposition for bone fractures. The basic features are based on secretion of proosteoblastic factors by tumors (cytokines and growth factors) that can tilt the normal bone remodeling toward abnormal bone formation state. It was reported that the OPG/RANKL system dictates the pathogenesis in a pure lytic and mixed metastatic bone lesions in which case elevation of RANKL to OPG ratio will be consistent with predominant osteolytic lesions [30, 44, 46].

### 3.1. Examples of Therapeutic Strategies to Prevent or Retard Progression of Bone Metastases

The best timing to initiate therapeutic intervention should be before local or distal metastases. Unfortunately, the majority of patients with lung cancer are diagnosed at stages where surgery and radiotherapy will be ineffective in curtailing metastases. It is very clear that optimization of the therapeutic outcome in lung cancer will require detailed understanding of the underlying pathways and molecular mechanisms of lung cancer metastases. Considering the poor prognosis of lung cancer after bone metastasis, the main goal of any therapeutic intervention should be to prevent or limit progression of bone metastases. This can be accomplished by implementing timely therapeutic interventions that could simultaneously target multiple steps involved in the tumor growth, migration, and metastasis as well as hampering tumor ability to invade metastatic sites. To be effective, therapeutic strategies should simultaneously impede the ability of cancer cells to invade local and distal sites while making the key metastatic sites unfertile (unconducive) for the invading tumor cells. With the overall goal of retarding progression of lung cancer and metastases, we highlight some of the strategies in literature that focused on impeding the ability of tumor to invade and those that exploit the crosstalk between tumor cells and bone microenvironment.

#### 3.1.1. Tumor-Based Strategies Based on Limiting Migration, Invasion, and Metastatic Potential

A common feature in lung cancer (just like other types of cancer) is the heterogeneity of the cell population which may be a product of varying degrees of gene alterations in the cell population and the impact of tumor microenvironment [47, 48]. In line with the heterogeneous feature is the understanding that in a tumor tissue not all the cells will possess metastatic capability to the same extent. In this regard, many investigators have paid close attention to a subgroup of cells that have self-renewal potential (cancer-stem cells, CSCs). CSCs have been identified in SCLCs and NSCLCs as possessing surface markers such as CD44, CD24, and ALDH [49]. Signaling pathways that have been implicated in regulating cancer-stem cell self-renewal include Wnt/*β*-catenin, Hedgehog, and Notch [50, 51]. However, it is not clear from literature whether only tumor cell subpopulation possessing self-renewal ability can metastasize. The point to note is that many studies that have focused on isolating and identifying distinct surface markers of CSCs have yielded molecular targets that have been implicated in conferring metastatic behaviors. An important contributing factor to tumor metastasis cascade is the detachment of cell through loss of cell adhesion molecules such as cadherins, integrins, and selectins. It is also important that dissemination of tumor cells and motility from primary to metastatic sites has been shown to involve epithelial-to-mesenchymal transition (EMT) whereby cell elongates and the extracellular matrix is degraded. EMT goes hand-in-hand with downregulation of epithelial markers in adherence junctions, tight junctions, and cytokeratin filament network (E-cadherin, occludins, type IV collagen, and laminin-1) [50, 52, 53]. Another feature of EMT is upregulation of mesenchymal markers such as N-cadherin, fibronectin, and fibroblast-specific protein 1. The discoveries that EMTs are induced by contextual signals, such as TGF-beta, EGF, FGFs, Wnt, and Notch ligands, have offered unique opportunities in lung cancer therapeutics. The interaction of tumor cells with the stoma is another important player of tumor cell detachment from the primary site with involvement of cancer related fibroblasts, tumor-associated macrophages, and endothelial cells [50]. Other regulatory molecular targets that can be exploited to retard tumor progression and metastasis are based on the fact that tumors require development of new blood and lymph vessels to grow. Key regulators of angiogenesis include cytokines, fibroblast growth factors, and vascular growth factor [54–56].

#### 3.1.2. Metastatic-Site Strategies Based on Limiting Tumor Interference with Bone Microenvironment

It is clear through accumulating evidence that the mere presence of tumor cells in blood circulation does not dictate the ability to survive at distant metastatic sites. The tumor cells in blood-stream and lymphatic system must withstand considerable amount of stresses. It has been reported that tumor cell extravasation and establishment of micrometastases will require key regulators like vascular endothelial growth factor (VEGF) and SDF-1/CXL12 that increase endothelial permeability at the metastatic sites [18, 57]. It was reported that CXCR4 ligand CXCL 12/SDF-1*α* is abundant in bone marrow stromal cells [57]. It was also shown in another study that CXR4 together with other factors such as CTGF, IL-11, and OPN promoted osteolytic bone metastases [58]. The migration of tumor cell across the basement membrane at metastatic site is not well understood but many investigators have implicated the plasminogen-activator system consisting of serine-protease plasmin [59–61].

Adaptation of tumor cells in bone microenvironment has received considerable amount of attention especially for breast and pancreatic cancers. The prevailing mechanism by which tumors survive at bone metastatic sites is still not fully understood. However, there are some interesting targets that are involved in tumor interference of normal bone hemostasis that are worthy of consideration in optimizing lung cancer treatment regimen. These include OPG/RANK/RANKL pathway, PTHrP, chemokines, and chemokines receptors. Reports from various studies have demonstrated increases in survival through application of agents that target bone responses to tumor such as bisphosphonates which reduce osteoclast bone resorption [62, 63]. These observations offered clues that responses of bone to the invading tumor cells should be targeted in specific treatments for bone metastases rather than focusing on the tumor alone (see Table 1).

## 4. Challenges, Conclusions, and Future Perspective

Bone metastases are a major clinical problem in lung cancer that is deserving continual attention. Compared to our knowledge of bone metastases in breast and prostate cancers, there is limited understanding of molecular mechanism of bone metastases in lung cancer. The goal of therapeutic interventions should not be limited to lessening the impact or reducing the cases of skeletal related events but to proactively retard the progression of lung cancer in a timely manner so as to improve survival. We strongly believe (as many other investigators would) that lung cancer prognosis will significantly improve if predisposition to invasive and metastatic behaviors can be detected in a timely fashion to guide therapeutic interventions. The field will benefit from ongoing efforts on developing new molecular markers that can potentially be applied in (i) identifying aggressive forms of lung cancer, (ii) predicting metastatic potential at early stages of the disease, (iii) predicting lung cancer aggressiveness and cases of relapse, (iv) detecting and monitoring individuals with benign pulmonary nodules from those with early malignancies, and (v) tailoring treatment regimen to the disease stages. Ideally, such optimized therapeutic strategies will address concurrently the multiple pathways that are involved in the progression of lung cancer from the primary sites, such as (a) local invasion of the lung tissues including the mediastinum and the chest wall, (b) gaining access to the lymphatic systems via regional lymph nodes, and (c) spreading of tumor into distant sites such as the liver, brain, and bone.

## Figures and Tables

**Table 1:** Examples of lung cancer therapeutic strategies that are based on bone metastases.

Experimental details	Treatment target	Observations
NSCLC patients with multiple bone metastases were treated with gefitinib.	Epidermal growth factor receptor tyrosine kinase inhibitor (EGFR-TKI)	Treatment halted progression of bone metastasis [13]

Screening samples from NSCLC patients obtained from 52 primary sites and 75 bone metastatic sites.	RANK/RANKL/OPG	Differential expressions of RANKL, RANK, and OPG were observed [32]

Preclinical studies after intratibial implantation of NSCLC cells in SCID mice.	EGFR and RANKL	Erlotinib, a EGFR-TKI, inhibited osteolytic bone invasion in SCID mice [13]

Screening of clinical specimens obtained from NSCLC patients.	Wnt/*β*-catenin	Elevated expression of Dickkopf-related protein 1 (DKK1) was observed. Differentiation of osteoblast was inhibited by DKK1 [9]

Preclinical studies of injecting NSCLC cells in SCID mice.	Colony stimulating factor (CSF1)	Suppression of CSF1 resulted in significant reduction in osteolytic lesions [16]

Ectopic expression of miR-33a in A549 cell lines.	PTHrP	miR-33a expression was inversely correlated with PTHrP [27]

100 patients with resectable NSCLC and asymptomatic bone metastases were treated with zoledronic acid (ZA) and/or strontium-89 (Sr-89).	Inhibition of bone resorption	Treatment with ZA and/or Sr-89 significantly extended the time for first SRE as well as survival time. Annual incidence of SREs was reduced [64]

Preclinical injection of NSCLC cells in athymic mice. The percentage osteolytic area of femur and tibia was evaluated.	Reduction of bone resorption	Treatment with ZA significantly reduced tumor-induced osteolysis [43]

220 NSCLC patients with skeletal metastases at time of diagnosis. The patients were treated with gefitinib.	EGFR-TI	Patients treated with EGFR-TKI had significantly longer survival and achieved overall 50% protective effect [65]

Preclinical studies of implantation of NSCLC cells in athymic mice.	EGFR-TI	Erlotinib inhibited tumor-induced osteolytic invasion in bone metastasis [10]

Preclinical studies of implantation of SCLC cells in SCID mice.	Anti-PTHrP neutralizing antibody	Suppression of osteoclast activity [44]

Preclinical implantation of SCLC cells in SCID mice.	Reveromycin A that targets isoleucyl-tRNA synthetase (IleRS)	Inhibiting osteoclast-apoptosis via suppression of IleRS in osteoclasts [44]
